# Millets for food and nutritional security in semi-arid Bundelkhand, India: historical, scientific, and socio-economic perspectives

**DOI:** 10.3389/fnut.2026.1759815

**Published:** 2026-05-26

**Authors:** Rumana Khan, Silpa Sahoo, Shivam Yadav, Jitendra Kumar Tiwari, Ajaya Kumar Rout, Nidhish Kumar, Manoj Kumar Singh, Dinesh C. Joshi, P. Sanjana Reddy

**Affiliations:** 1 Department of Genetics and Plant Breeding, Rani Lakshmi Bai Central Agricultural University, Jhansi, India; 2 Department of Genetics and Plant Breeding, GB Pant University of Agriculture and Technology, UdhamSingh Nagar, India; 3 Indian Council of Agricultural Research (ICAR)- Vivekananda Parvatiya Krishi Anusandhan Sansthan, Almora, India; 4 Indian Council of Agricultural Research (ICAR) – Indian Institute of Millets Research (IIMR), Hyderabad, India

**Keywords:** biofortified varieties, Bundelkhand, food security, millets, nutritional security, policies

## Abstract

Millets are ancient cereal grains cultivated for over 10,000 years across more than 90 countries. They are nutritionally rich, providing carbohydrates, high-quality protein, fiber, vitamins, and micronutrients (such as iron and zinc), making them crucial for food and nutritional security. Despite their intrinsic nutritional and ecological value, the total area under millet cultivation declined substantially after the mid-20th century Green Revolution, as government-backed agricultural policies largely incentivized cereal based agronomy. Central India's Bundelkhand region faces food and nutrition insecurity due to erratic rainfall, low soil fertility, land degradation, frequent droughts, and other natural calamities. In this challenging context, the systematic revitalization of millet cultivation is increasingly recognized as a critical intervention. Historically integral to Bundelkhand's traditional farming systems, these indigenous crops offer a reliable climate-resilient alternative to water-intensive major cereals. Globally, millet production is about 30 million tons (with an average yield of ~1 t/ha), of which India contributes roughly 40%−43% (approximately 16.4 mt from 13.3 million hectares). However, millet cultivation in Bundelkhand lag significantly below the national average in terms of net cultivated area, production and productivity. This review outlines the historical trajectory of millet cultivation in Bundelkhand, its decline in post-Green Revolution era, and its emerging role in ensuring food and nutritional security. The review also explores recent advances in millet improvement research, specifically highlighting the development of high-yielding, stress-tolerant, and biofortified varieties. Furthermore, their nutritional benefits, the efficacy of supportive public policies, and the impact of community-driven revival initiatives. By integrating agronomic, genetic, and socio-economic perspectives, the review highlights millet's potential to diversify cropping systems, restore degraded agro-ecosystems, and strengthen the livelihoods of smallholder farmers and resilience in climate-vulnerable regions like Bundelkhand, thus contributing to sustainable, resilient, and equitable food and nutrition systems.

## 1 Introduction

Global food and nutrition security is under severe strain from population growth, climate change, and persistent malnutrition, especially in developing countries. Intensive cereal monocropping has further undermined this system by depleting soil health and resilience. To address these issues, there is increasing agreement among scientists and policymakers that urgent action is needed to diversify the food system by revitalizing traditional, climate-smart crops that were overlooked during the Green Revolution in India (1). Millets, a diverse group of coarse grains (2), are central to this effort, offering a balanced nutritional profile, low input requirements, and the ability to thrive in adverse environments. They are often hailed as “future crops” for sustainable and health-focused agriculture, especially in arid and semiarid tropical regions (3). Based on grain size millets are broadly classified into major and minor millets. Major millets include pearl millet (*Pennisetum glaucum*), sorghum (*Sorghum bicolor*), and finger millet (*Eleusine coracana*), and minor millets include foxtail millet (*Setaria italica)*, barnyard millet (*Echinochloa* spp.), proso millet (*Panicum miliaceum)*, kodo millet (*Paspalum scrobiculatum*), and little millet *(Panicum sumatrense)*. FAO and several governments have recently designated millets as climate-resilient crops, recognizing their role in tackling hunger and environmental stress. The Bundelkhand region of central India, covering 14 districts, seven each in Uttar Pradesh and Madhya Pradesh. Farmers in the Bundelkhand region face numerous environmental challenges, such as unpredictable rainfall, frequent, prolonged droughts, and erosion-prone, nutrient-deficient soil (4, 5). These factors collectively severely affect crop production and productivity (6, 7). Agriculture in this region is predominantly rain-fed, serving as the primary livelihood for approximately 60% of the local population. Nevertheless, the increasing unpredictability of climate, has substantially constrained agricultural productivity. Consequently, farm-dependent families have been forced to migrate, while food insecurity has become a recurring regional problem rather than an occasional crisis. Millets, which historically formed the backbone of Bundelkhand's food security, are well-suited to the region's harsh agroecology and have served as a resilient safeguard. However, the shift to input-intensive crops, such as wheat and rice, after the Green Revolution has systematically weakened the socio-ecological system that supported millets. Despite their marginalization, millets remain highly suitable for the region's agroecological conditions.

Unfortunately, the revival of millets faces barriers such as limited financial support, the unavailability of improved high-yield varieties (8, 9), weak extension services, and marketing hurdles (10, 11).

Therefore, reviving millets in the Bundelkhand region will require more than just restoring traditional agriculture; it demands an innovative, integrated approach that draws lessons from history, science, and socioeconomic realities. The present review brings together historical, nutritional, agronomic, and policy evidence to re-evaluate millet's role in the nutritional and economic security of Bundelkhand. Leveraging integrated evidence, we propose a tripartite strategy to rebuild the millet value chain: (1) Crop improvement to develop and disseminate high-performing, locally adapted millet varieties; (2) Value addition to expand processing and product development results in improved profitability and nutrition; and (3) Market and policy support to strengthen market linkages and enact policies that ensure market neutrality for millet farmers. Based on these objectives, the review outlines how millets can be repositioned from “lost crops” to keystones of a climate-resilient, nutrition-secure agriculture in Bundelkhand.

## 2 Nutritional composition of millets and health benefits

**Figure 1:**
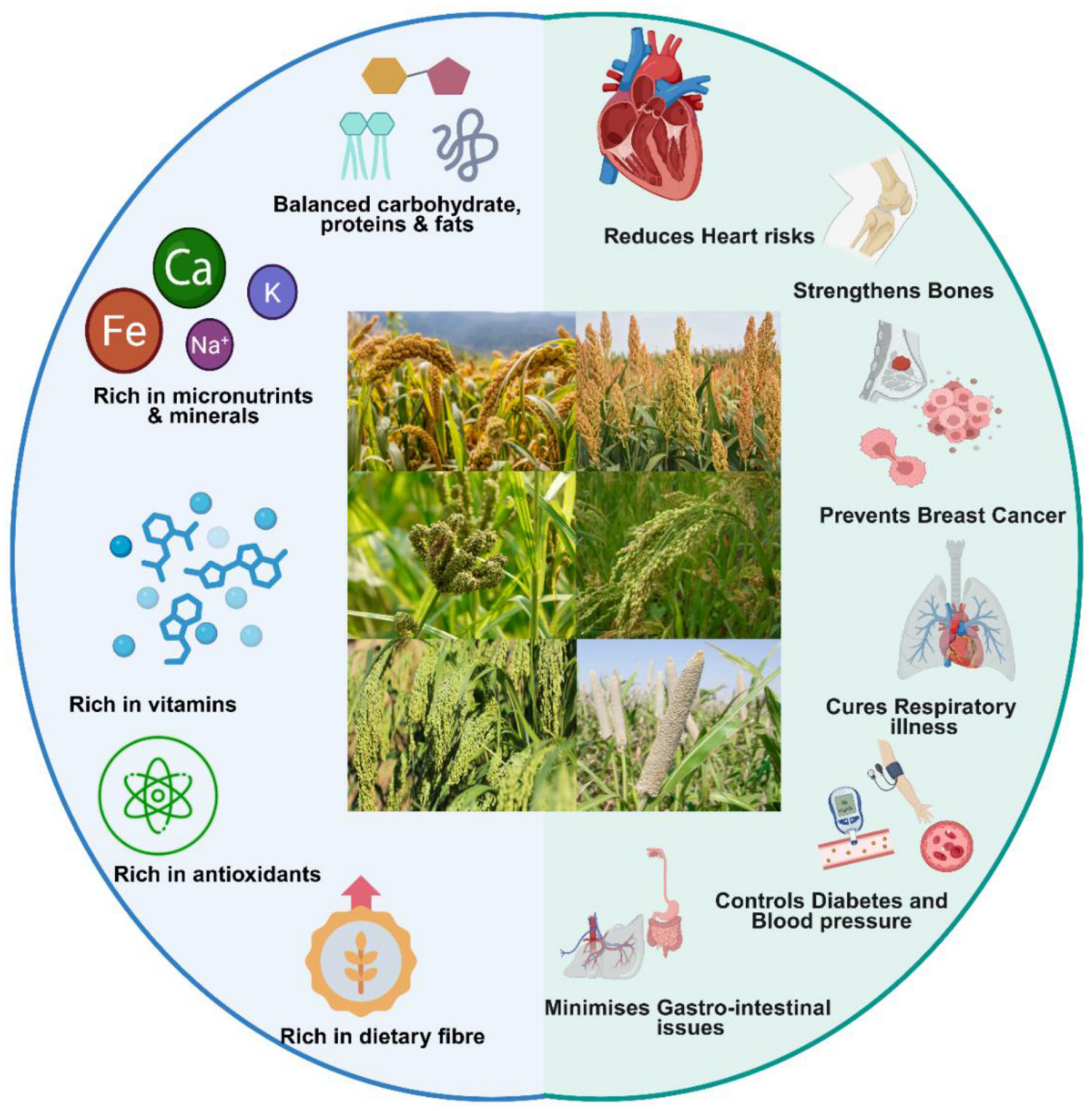
Summary of different nutritional aspects of millets and their associated health benefits.

The Bundelkhand region suffers from nationwide most severe rates of malnutrition. Recent demographic surveys indicate that approximately 40–45% of children under five years of age are underweight, and anemia among women is common, reflecting deficiencies in essential dietary micronutrients (12, 13). Similarly, one out of four adults was suffering from chronic energy deficiency, estimates suggest that over 80% of Bundelkhand's total population suffers from some form of anaemia (14). The National Family Health Survey-5 (NFHS-5) reports 40.2% stunting among children under five and 59.1% anemia among women ages 15–49, both driven by socioeconomic and environmental stressors (15). Similar vulnerabilities are observed in nearby tribal communities, highlighting food insecurity and ecological constraints (16). Millets, often termed traditional “nutri-cereals,” can offer a nutritionally strategic solution to directly address these deficits. Millets typically contain ~60%−75% complex carbohydrates, ~7%−12% protein, and 2%−5% fat, with 2%−7% dietary fiber. Moreover, millets provide essential minerals and vitamins, enhancing their nutritional value. They are especially high in dietary fiber, have a low glycemic index (17), and are excellent sources of essential micronutrients, such as calcium, iron, zinc, phosphorus, and B-complex vitamins (thiamine, riboflavin, niacin, and folic acid). Among millets, finger millet stands out with exceptionally high calcium content (364 mg/100 g), whereas barnyard and little millets provide significant iron and fiber. Compared to rice and wheat, millet proteins are more balanced in essential amino acids, including lysine, methionine, and cysteine. Furthermore, millets exhibit notable mineral diversity–foxtail, finger, and little millets are particularly rich in micronutrients (Figure 1) like calcium (10–348 mg/100 g), iron (2.2–17.7 mg/100 g), zinc (0.4–2.8 mg/100 g), and phosphorus (189–293 mg/100 g); and vitamins such as thiamine (0.15–0.60 mg/100 g), niacin (0.89–4.6 mg/100 g), and riboflavin (0.9–0.28 mg/100 g) (18, 19) (Table 1). Millets also contain health-promoting phytochemicals and antioxidants that impart therapeutic properties for various disorders and diseases, thereby conferring nutraceutical value (20). Regular, consumption of millets may help in managing metabolic and lifestyle-related disorders such as diabetes, obesity, high cholesterol, and cardiovascular diseases, while also supporting bone health, gut health, and colon function (Figure 1) (21–24). Their high nutritional value, climate resilience, and adaptability to marginal conditions make millets a sustainable and health-friendly alternative to major grain cereals.

**Table 1:** Nutrient composition of selected major and minor millets, in comparison to staple cereals per 100 g edible portion.

Component(s)	Major millets		Minor millets						Major cereals	
	Sorghum	Pearl-millet	Finger millet	Kodo millet	Proso millet	Foxtail millet	Little millet	Barnyard millet	Wheat	Rice
Carbohydrates (g)	67.7	61.8	66.8	66.2	70.4	60.1	65.5	65.5	64.7	78.2
Protein (g)	9.9	10.9	7.2	8.2	12.5	12.3	10.1	05.2	10.6	07.9
Fat (g)	1.73	5.43	1.92	2.55	1.1	04.3	03.89	02.2	1.47	0.52
Energy (Kcal)	334	347	320	331	341	331	346	307	321	356
Dietary fiber (g)	10.2	11.5	11.2	8.4	7.2	7.8	7.7	8.2	11.2	02.8
Ca (mg)	27.6	27.4	364	15.3	14	31	16.1	20	39.4	07.5
P (g)	274	289	210	101	206	188	130	280	315	96.0
Mg (mg)	133	124	146	122	153	81	91	82	125	19.0
Zn (mg)	1.9	2.7	2.5	1.6	1.4	2.4	1.8	3	2.80	01.2
Fe (mg)	3.9	6.4	4.6	2.3	0.8	2.8	1.2	5	3.90	0.6
Thiamine (mg)	0.35	0.25	0.37	0.29	0.41	0.59	0.26	0.33	0.46	0.05
Riboflavin (mg)	0.15	0.21	0.19	0.09	0.11	0.11	0.09	0.09	0.10	0.05
Niacin (mg)	2.1	0.9	1.3	1.5	4.5	3.2	1.3	4.2	2.70	1.70
Folic acid (μg)	39.4	36.1	34.7	39.5	–	15	36.2	–	30.1	9.32

Source: Anitha et al. (118); Longvah et al. (119).

To incorporate millets in the diet, modern interventions, such as Integrated Child Development Services (ICDS), face cultural and logistical barriers (25). Conversely, indigenous nutritional practices, embedded in IKS, utilize drought-tolerant millets, pulses, and wild greens, offering a culturally resonant solution (26, 27). Revitalizing millet-based food systems, as highlighted by traditional recipes and culinary heritage in Bundelkhand, can enhance nutritional security and resilience to climate challenges in the region (28).

## 3 Scenario of millet cultivation in India

**Figure 2:**
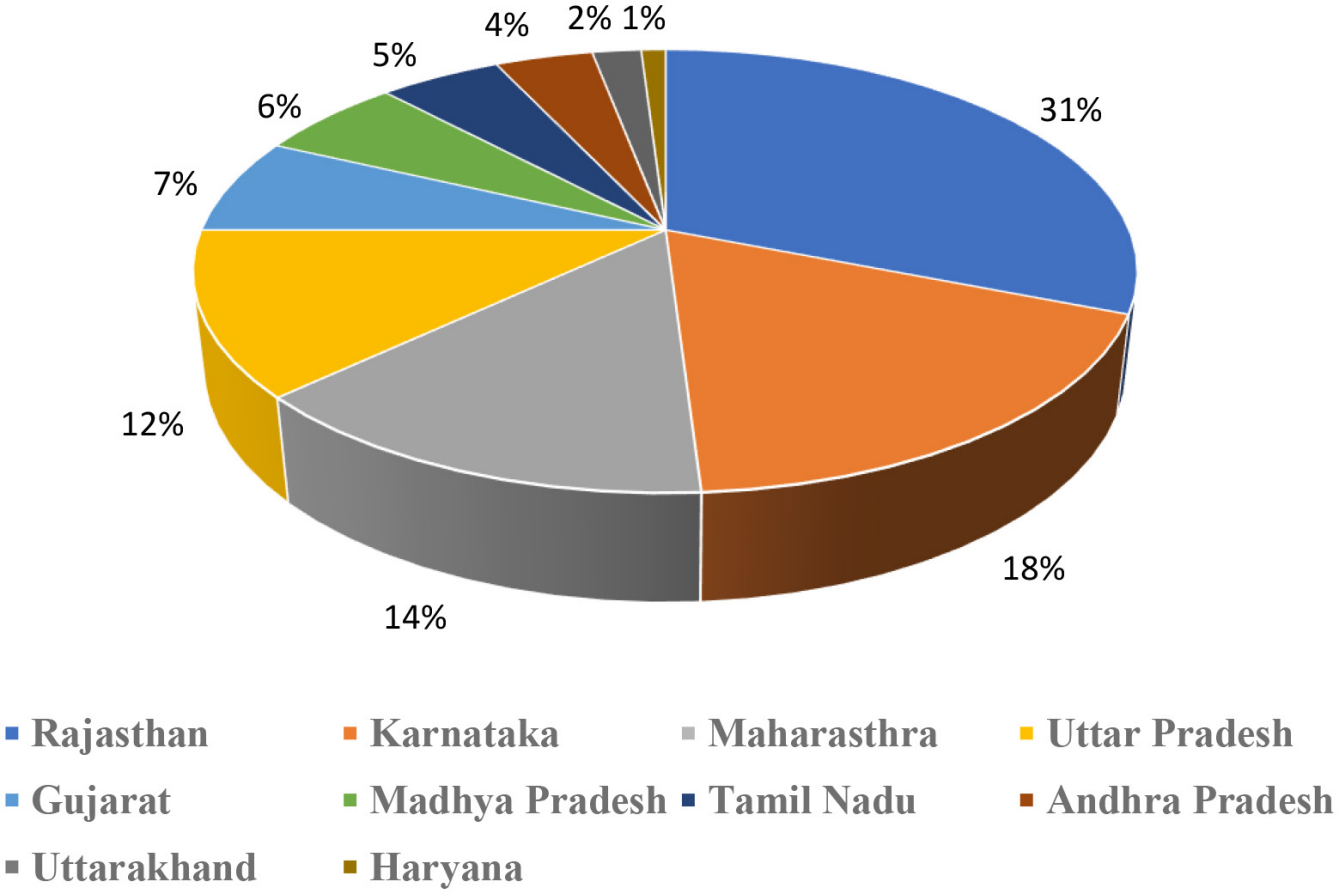
State-wise share of millet production in India during 2024–25. Rajasthan accounted for the largest share of national millet production (31%), followed by Karnataka (18%), Maharashtra (14%), and Uttar Pradesh (12%), indicating a strong concentration of millet production in drought-prone and semi-arid states.

India produces about 20% of the world's millet, ranking as the top producer, followed by Niger (13%), China (5%), and Sudan (3%) in global millet production and trade (29). A 25-year study of millet production and productivity in India shows a sharp decrease in cultivated area but an increase in both output and efficiency (Table 2) (2). In the 2023–24 agricultural year, millet production reached 17.57 million metric tons (MMT) from a cultivated area of 13.09 million hectares (hereafter mha), with an average yield of 1,342 kg per hectare (30). While national statistics offer a broad overview, assessing production at the state level is necessary to understand the distribution and spatial patterns of cultivation in India. Millet cultivation is unevenly distributed, mainly concentrated in a few states with arid and semi-arid climates. Rajasthan leads with nearly 4.77 mha, followed by Maharashtra with 2.29 mha, Karnataka with 1.72 mha, and Uttar Pradesh with 1.35 mha dedicated to millet cultivation. Production follows the trend of area under millet cultivation, with Rajasthan as the leading producer with 4.89 MMT, accounting for nearly 31% of India's total millet output. Karnataka follows with 2.70 MMT (18%), while Maharashtra and Uttar Pradesh each contributed 14% and 12%, respectively (Figure 2). These four states together account for roughly 75% of India's total millet production, underscoring their key role in the millet supply chain (31).

**Table 2:** Trends in area (‘000 ha), production ('000 tons), and productivity (t/ha) of millets in India.

Period	Bajra			Jowar			Ragi			Small millets		
	Area	Production	Productivity	Area	Production	Productivity	Area	Production	Productivity	Area	Production	Productivity
TE 1968-69	12366	4,485	0.36	18,403	9,692	0.53	2,171	1,721	0.79	4729	1,733	0.37
TE 1973-74	12508	5,589	0.45	16,335	7,929	0.49	2,371	2,068	0.87	4437	1,729	0.39
TE 1983-84	11520	6,131	0.53	16,469	11,578	0.7	2,527	2,672	1.06	3641	1,514	0.42
TE 1993-94	10183	6,173	0.61	12,704	10,773	0.85	1,973	2,570	1.3	1986	889	0.45
TE 2003-04	9294	8,371	0.9	9,475	7,084	0.75	1,576	1,885	1.2	1234	533	0.43
TE 2013-14	7962	9,423	1.18	2,441	2,842	1.16	1,167	1,829	1.57	745	439	0.59
TE 2022-23	7316	10,604	1.45	3,952	4,318	1.09	1,155	1,766	1.53	448	370	0.83
TE 2023-24	7375	10,716	1.45	4,076	4,737	1.16	1215	1,670	1.37	480	449	0.93
TE 2024-25	6835	9,490	1.39	4,302	5,265	1.22	1162	1,692	1.45	413	386	0.93
CAGR (%)	−11.1	13.9	24.9	−23.3	−15.1	14.5	−11.2	−3.0	10.7	−41.2	−23.5	15.5

CAGR denotes the compound annual growth rate in per cent, and TE refers to the triennium ending.

**Figure 3:**
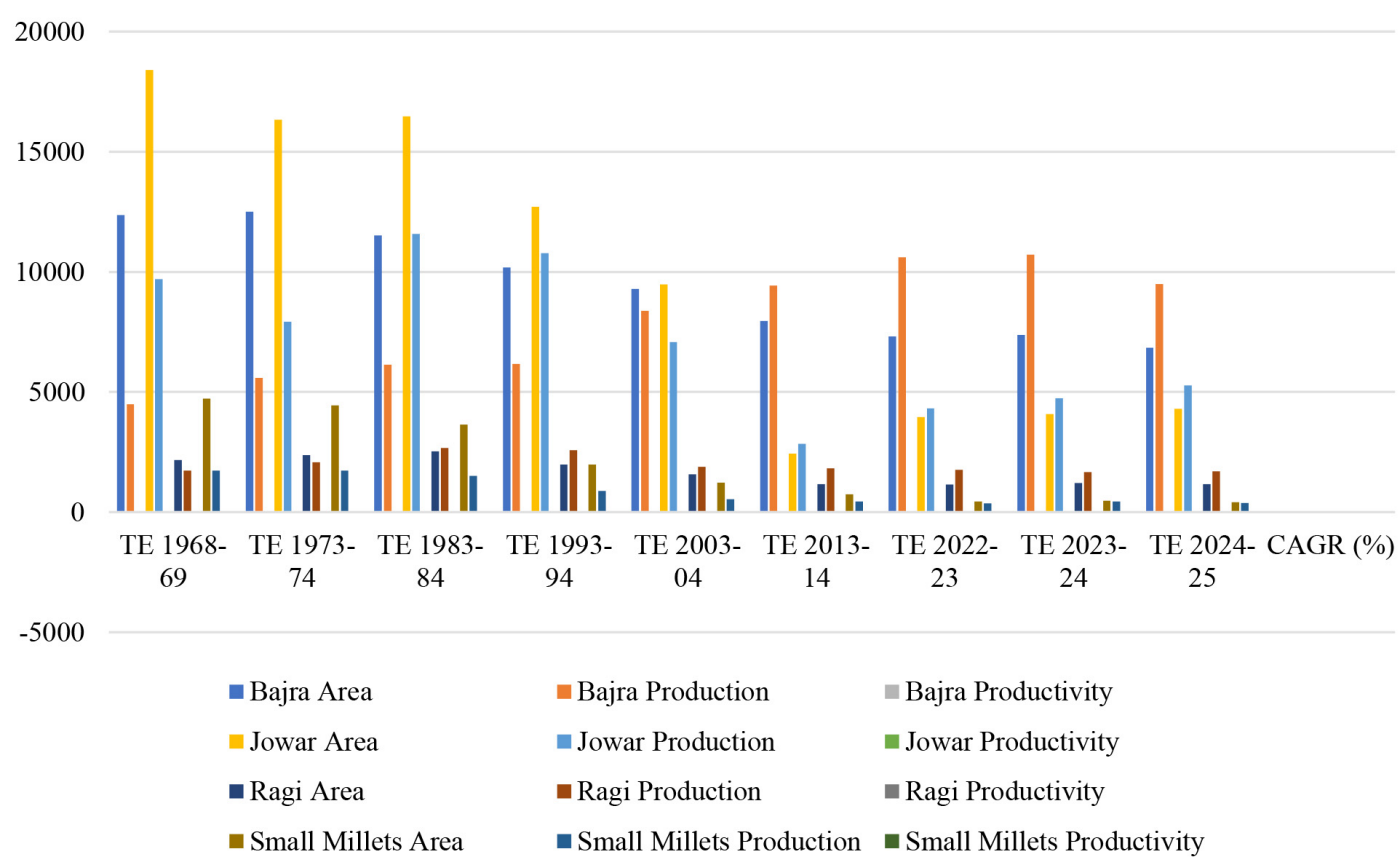
Long-term trends in area, production, and productivity (y-axis) of major millets in India across triennium-ending periods from 1968–69 to 2024–25 with compound annual growth rate (CAGR) (x-axis). Source: Department of Agriculture & Farmers Welfare, UPAg Portal (131).

An analysis of the 56-year trend from the triennium ending (TE) 1968-69 to TE 2024-25 on the area, production, and productivity of millets is shown in Table 2 and Figure 3. The growth rate analysis revealed negative growth for all millets, with small millets experiencing the largest decline, followed by jowar and ragi. The two crops, bajra and ragi, showed increases in production to 5.00 million tons and 0.29 lakh tons, respectively, during this period, mainly due to rising productivity levels over the years. Productivity increased from 0.36 to 1.39 t/ha for bajra (with a CAGR of 24.9%), from 0.79 to 1.45 t/ha for ragi (CAGR of 10.7%), and from 0.37 to 0.93 t/ha for small millets (CAGR of 15.5%).

The increase in production and productivity of pearl millet and sorghum has prompted systematic research on the availability of suitable male-sterile lines, exploitation of heterosis, and development of high-yielding hybrids, as well as varieties suitable for different agro-climatic zones of India. Finger millet, a major millet grown in many parts of India, also benefited from focused research on developing high-yielding varieties by a planned hybridization program along with mutation breeding for development of early-maturing types as well as development of complete and partial male sterile lines for practical hybrid development. Major research and development activities are insufficient for remaining minor millets; therefore, their area, production, and productivity declined.

**Figure 4:**
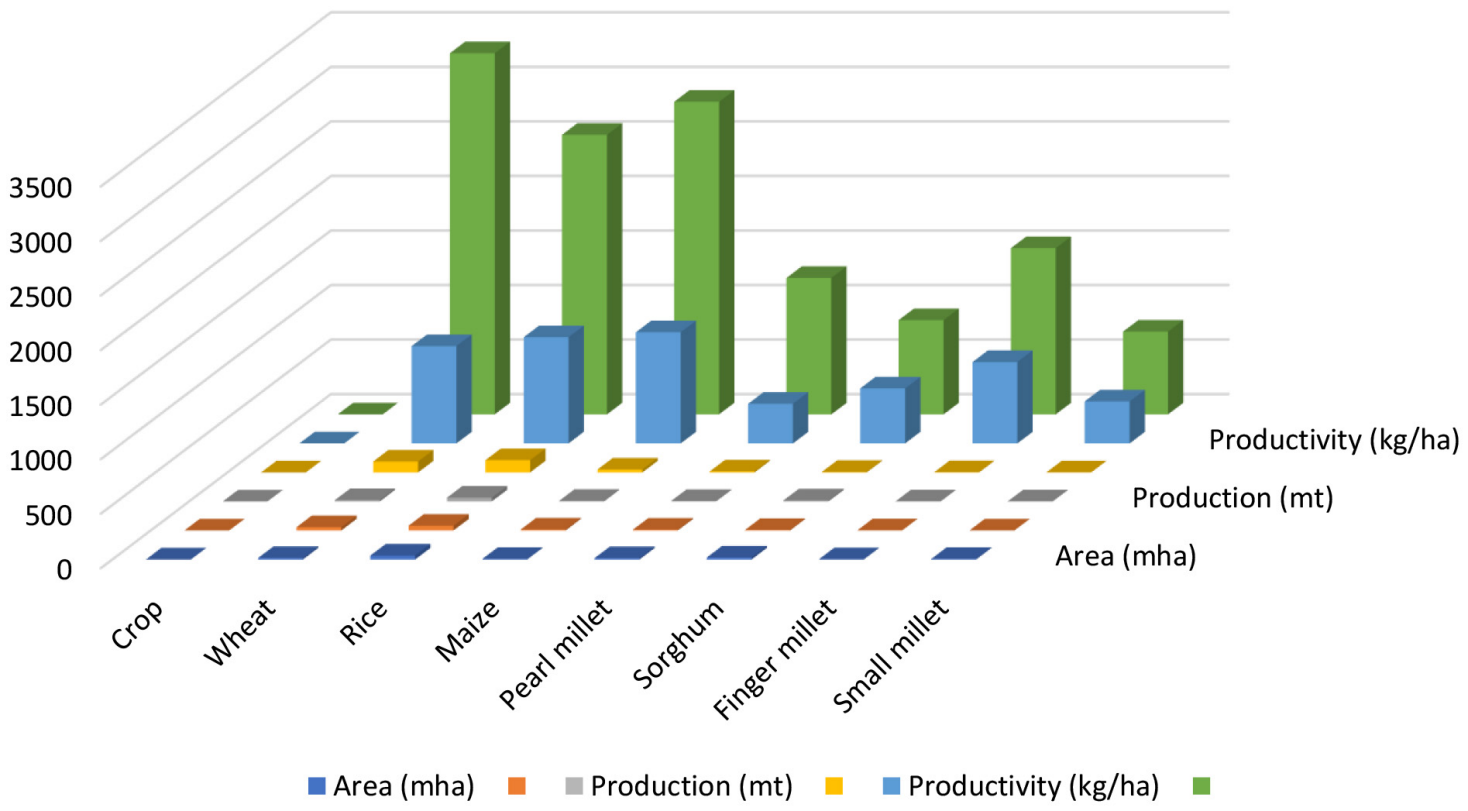
Trends of area, production, and productivity of major millets with wheat, rice, and maize in India. The figure highlights the relatively lower cultivated area and production of millet crops compared with major cereals, while productivity varies substantially among crops.

A comparative analysis of millets vs. major cereals like wheat, rice, and maize shows a significant increase in area and production for these cereals, along with a sharp decrease in area and production for millets (Table 3, Figure 4).

**Table 3:** Area, production, and productivity of millets, in comparison to major cereals (wheat, rice and maize) in India.

Crop	Area (mha)		Production (mt)		Productivity (kg/ha)	
	**1963–67**	**2015–20**	**1963–67**	**2015–20**	**1963–67**	**2015–20**
Wheat	13.5	30.30	12.1	100.31	892	3,311
Rice	35.9	43.84	35	112.36	974	2,563
Maize	4.9	9.31	5	26.72	1,019	2,866
Pearl millet	12	7.34	4.4	9.19	363	1,251
Sorghum	18.1	5.10	9.1	4.36	504	864
Finger millet	2.41	1.06	1.77	1.63	746	1,524
Small millet	4.64	0.53	1.79	0.39	385	759

Similarly, the share of millets in the total food grain area has gone down from 31.71% in TE 1968–69 to 9.84% in TE 2022–23. The corresponding decrease in production is 20.04% to 5.34% (Table 4).

**Table 4:** Long-term changes in the contribution of millets to total foodgrain area and production in India based on triennium-ending (TE) averages, 1968–69 to 2022–23.

Period	Bajra	Jowar	Ragi	Small millets	Total	Bajra	Jowar	Ragi	Small millets	Total
Share in food grains area (%)						Share in food grains production (%)				
TE 1968-69	10.4	15.5	1.83	3.98	**31.71**	5.11	11	1.96	1.97	**20.04**
TE 1973-74	10.2	13.3	1.9	3.6	**25.4**	5.46	7.75	2.02	1.69	**16.92**
TE 1983-84	9.0	12.8	2.0	2.8	**26.6**	4.43	8.36	1.93	1.09	**15.81**
TE 1993-94	8.3	10.4	1.6	1.6	**21.9**	3.38	5.89	1.41	0.49	**11.17**
TE 2003-04	7.7	7.9	1.6	1.0	**18.2**	4.18	3.54	0.94	0.27	**8.93**
TE 2013-14	6.45	1.98	0.94	0.6	**9.97**	3.62	1.09	0.7	0.17	**5.58**
TE 2022-23	5.6	3.02	0.88	0.34	**9.84**	3.32	1.35	0.55	0.12	**5.34**

The decline in millet production relative to major cereals (wheat, rice, maize) is driven by post-Green Revolution policies prioritizing high-yield staples, lack of minimum support prices (MSP), and high processing costs for millets. Conversely, better subsidies, irrigation, and market demand incentivized farmers to switch to rice and wheat (120).

## 4 History and scenario of millets cultivation in Bundelkhand

### 4.1 The Bundelkhand region

**Figure 5:**
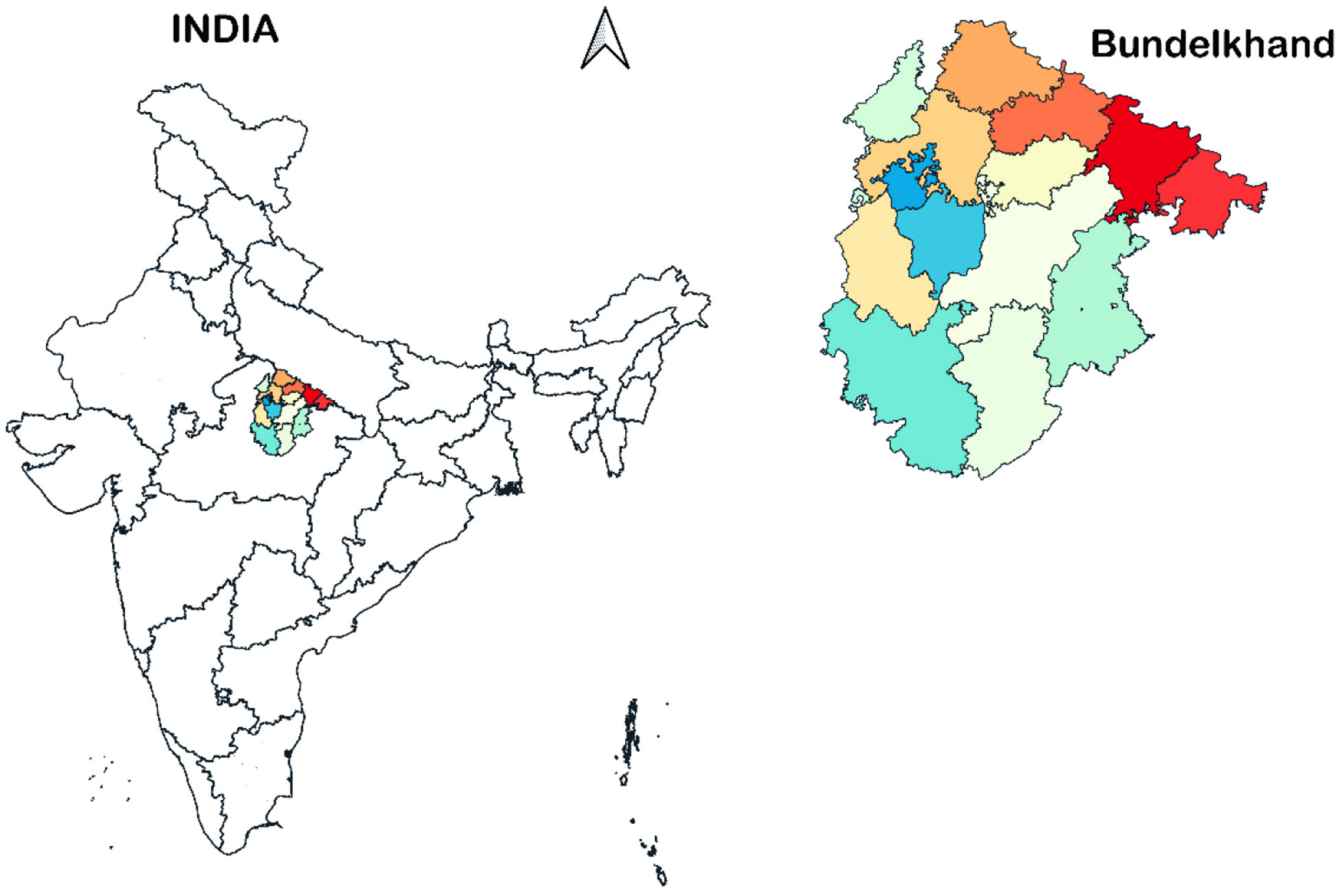
Geographical location of Bundelkhand in central India, showing the region across southern Uttar Pradesh and northern Madhya Pradesh. The highlighted districts represent the Bundelkhand agro-ecological zone considered in the present study.

The Bundelkhand region includes seven districts from Uttar Pradesh—Banda, Chitrakoot, Hamirpur, Jalaun, Jhansi, Lalitpur, and Mahoba—as well as seven districts from Madhya Pradesh: Chhatarpur, Datia, Damoh, Panna, Sagar, Niwari, and Tikamgarh (Figure 5). Despite sharing a common cultural heritage and traditions, the region was divided after independence based on recommendations from the States Reorganization Commission. Located in north-central India, Bundelkhand spans Uttar Pradesh and Madhya Pradesh, lying between the Indo-Gangetic Plain to the north and the Vindhya Range to the south. Its northern boundary is marked by the Yamuna River, and the Sind River forms its western edge. The Narmada flows near its southern boundary, close to Sagar district. However, these rivers are not central to the region's economy. Instead, the main rivers that influence Bundelkhand's economy flow from south to north and include the Ken, Betwa, and their tributaries, along with several other streams, the most significant of which is the Baghain.

Due to its geographic location, the region is vulnerable to drought and experiences irregular, low rainfall. This has made agriculture heavily dependent on the monsoons, often leading to crop failures and hardships for farmers (32). Due to several ecological constrains, the Bundelkhand region has limited irrigation, large areas of wasteland, lower rainfall, and poorer soil quality than the state averages in Uttar Pradesh and Madhya Pradesh (33–35). Recently, the region has faced many challenges, including poverty, drought, and underdevelopment (36). It is one of the most severely degraded ecosystems, characterized by rugged terrain, heavily eroded and fragmented land, depleted soil fertility, and low water retention capacity.

### 4.2 History of millet cultivation in Bundelkhand

Before the introduction of high-yielding cereal varieties, millets were the backbone of the food and agriculture system. Bundelkhand's tradition and cropping system included a variety of coarse grains, especially sorghum (jowar), pearl millet (bajra), and various small millets like kodo millet (kodo), little millet (kutki or sami), barnyard millet (sawa), and finger millet (ragi or madua). These crops were not only sources of sustenance during repeated droughts but also part of the region's cultural heritage. During agricultural crises, these grains provided essential support. Socio-economic reports from the 2015 agrarian crisis indicated that at least 17% of families survived solely by consuming “ghas ki roti” (colloquially translated as grass bread). However, agronomically, this term actually refers to *fikar* (foxtail millet) and samai (little millet), which are highly nutritious, indigenous, and drought-resilient traditional grains of Bundelkhand region (132, 137). Beyond statistics, the story emphasizes millet's role in regional food security, a role that has waned over time.

### 4.3 Scenario of millets cultivation in Bundelkhand

In Bundelkhand, the main challenges in agriculture include water scarcity, depletion of natural resources, and low crop yields (1–1.5 q/ha). The region has a low rainwater-use efficiency of 35%−45% and faces issues, such as erosion, poor soil fertility, frequent droughts, insufficient irrigation, and limited vegetation cover. It experiences extreme temperature fluctuations, with summer heat exceeding 45 °C and winter temperatures dropping to nearly 1 °C. Rainfall occurrence and distribution are also unpredictable. A comprehensive drought-risk assessment identified eight districts of Bundelkhand experience moderate to severe vulnerability to drought (34). In recent decades, millet cultivation has declined due to several factors, including the shift toward cash crops, lack of market infrastructure, and changing dietary preferences. Nonetheless, there is increasing recognition of the importance of revitalizing millet farming to improve food security, nutrition, and climate resilience.

A 56-year trend analysis shows that the area under millet cultivation has significantly decreased in India, including in the states of Uttar Pradesh and Madhya Pradesh. Despite this, pearl millet production has risen considerably across India (136.42%), UP (274.11%), and MP (421.57%). Conversely, sorghum (jowar) and small millet production have fallen (Table 5). The Bundelkhand region has very low productivity for all millets compared to national and state averages (Table 6). Over the past 4 years, district-wise data from Bundelkhand UP reveal that Banda district experienced the highest growth in pearl millet area (44%), production (456%), and yield (1%), while other districts saw declines. In districts such as Hamirpur, Jalaun, Jhansi, Lalitpur, and Mahoba, the area under sorghum production and productivity has increased. Lalitpur district recorded a sharp increase in the area, production, and productivity of small millets (Table 7). Similarly, in Bundelkhand, MP, districts Datia and Sagar have seen increases in pearl millet area, production, and productivity over the last 4 years. Sagar district also reported increased sorghum production, as did Panna for small millets (Table 8).

**Table 5:** Long-term changes in area and production of selected millets in India, Madhya Pradesh, and Uttar Pradesh, based on triennium-ending (TE) averages for 1968–69 and 2022–23.

Crops	Area (000 ha)		Change (%)	Production (000, tons)		Change (%)
	**TE 1968-69**	**TE 2022-23**		**TE 1968-69**	**TE 2022-23**	
India						
Bajra	12,366.33	7,315.89	−40.84	4,485.00	10,603.56	136.42
Jowar	18,402.60	3,952.17	−78.52	19,691.87	4,317.68	−55.45
Ragi	2,171.20	1,154.69	−46.82	1,720.93	1,765.63	2.60
Small millets	4,729.13	447.66	−90.53	1,733.13	370.49	−78.62
MP						
Bajra	278.10	344.67	23.94	163.13	850.86	421.57
Jowar	2,504.00	111.00	−95.57	1759.00	217.49	−87.64
Ragi	18.27	-	-	4.40	-	-
Small millets	1,624.17	106.00	−93.47	321.27	92.19	−71.30
UP						
Bajra	1,041.83	906.67	−12.97	528.90	1978.66	274.11
Jowar	875.93	176.33	−79.87	426.93	272.61	−36.15
Ragi	186.20	-	-	129.93	-	-
Small millets	569.90	9.00	−98.42	339.10	6.56	−98.06

Source: Ministry of Agriculture and Farmer's Welfare (121); Ramadas et al. (122).

**Table 6:** Comparative status of millets area, production, and productivity in India, Uttar Pradesh, Madhya Pradesh, and the Bundelkhand region during crop year 2022–23.

Region	India			Uttar Pradesh			Madhya Pradesh			Bundelkhand		
	Area (mha)	Production (mt)	Productivity (kg/ha)	Area (mha)	Production (mt)	Productivity (kg/ha)	Area (m ha)	Production (mt)	Productivity (kg/ha)	Area (m Ha)	Production (mt)	Productivity (kg/ha)
Sorghum	5.23	4.64	887	0.5	0.45	900	0.4	0.35	875	0.3	0.2	667
Pearl millet	6.93	9.08	1,310	0.6	0.65	1,083	0.55	0.6	1,091	0.45	0.5	1,111
Finger millet	1.19	1.98	1,660	0.15	0.2	1,333	0.12	0.18	1,500	0.1	0.15	1,500
Foxtail millet	0.8	0.45	563	0.05	0.03	600	0.04	0.02	500	0.05	0.03	600
Barnyard millet	0.44	0.19	432	0.03	0.01	333	0.02	0.01	500	0.03	0.01	333
Total	14.59	16.34	1,120	1.33	1.34	1,008	1.13	1.16	1027	0.93	0.89	957

mha, million hectares, mt, million ton.

Source: Ministry of Agriculture and Farmer's Welfare (121).

**Table 7:** District-wise distribution of millets area, production, and productivity in the Bundelkhand region of Uttar Pradesh.

District	Year	Bajra			Jowar			Small millets		
		Area	Production	Yield	Area	Production	Yield	Area	Production)	Yield
Banda	2019–2020	3,268	5,989	1.83	22,404	41,439	1.85	21	15	0.71
	2022–2023	4,712	8,665	1.84	20,777	40,390	1.94	7	5	0.71
	**% change**	**44%**	**45%**	**1%**	**−7%**	**−3%**	**5%**	**−67%**	**−67%**	**0%**
Chitrakoot	2019–2020	10,810	19,969	1.85	18,212	28,103	1.54	-	-	-
	2022–2023	11,115	15,039	1.35	17,144	21,910	1.28	349	226	0.65
	**% change**	**3%**	**−25%**	**−27%**	**−6%**	**−22%**	**−17%**	**-**	**-**	**-**
Hamirpur	2019– 2020	514	949	1.85	13,049	14,727	1.13	-	-	-
	2022–2023	89	133	1.49	16,105	24,657	1.53	-	-	-
	**% change**	**−83%**	**−86%**	**−19%**	**23%**	**67%**	**35%**	**-**	**-**	**-**
Jalaun	2019– 2020	14,663	27,727	1.89	5,471	7,689	1.41	-	-	-
	2022– 2023	14,471	17,959	1.24	5,496	8,755	1.59	-	-	-
	**% change**	**−1%**	**−35%**	**−34%**	**0%**	**14%**	**13%**	**-**	**-**	**-**
Jhansi	2019– 2020	23	43	1.87	799	787	0.98	23	18	0.78
	2022– 2023	7	10	1.43	2,050	3,266	1.59	-	-	-
	**% change**	**−70%**	**−77%**	**−24%**	**157%**	**315%**	**62%**	**-**	**-**	**-**
Lalitpur	2019–2020	-	-	-	209	206	0.99	8	5	0.63
	2022– 2023	-	-	-	215	342	1.59	55	42	0.76
	**% change**	**-**	**-**	**-**	**3%**	**66%**	**61%**	**588%**	**740%**	**21%**
Mahoba	2019– 2020	28	52	1.86	2,960	2,114	0.71	15	11	0.73
	2022– 2023	4	6	1.5	3,674	5,915	1.61	6	4	0.67
	**% change**	**−86%**	**−88%**	**−19%**	**24%**	**180%**	**127%**	**-60%**	**−64%**	**−8%**

Source: Ministry of Agriculture and Farmer's Welfare (121). % change values are indicated in bold.

**Table 8:** District-wise distribution of millets area, production, and productivity in the Bundelkhand region of Madhya Pradesh.

District	Year	Bajra			Jowar			Small millets		
		Area	Production	Yield	Area	Production	Yield	Area	Production	Yield
Chhatarpur	2019–2020	14	8	0.57	6,086	6,427	1.06	1	-	0
	2022–2023	12	8	0.67	3,510	4,739	1.35	180	140	0.78
	**% change**	**−14%**	**0%**	**18%**	**−42%**	**−26%**	**27%**	**17,900%**	**-**	**-**
Damoh	2019–2020	3	6	2	45	30	0.67	1	-	0
	2022–2023	-	-	-	18	34	1.89	-	-	-
	**% change**	**-**	**-**	**-**	**−60%**	**13%**	**182%**	**-**	**-**	**-**
Datia	2019–2020	2,610	3,485	1.34	1,331	1,744	1.31	-	-	-
	2022–2023	4,774	5,423	1.14	3,489	4,571	1.31	-	-	-
	**% change**	**83%**	**56%**	**−15%**	**162%**	**162%**	**0%**	**-**	**-**	**-**
Niwari	2020–2021	-	-	-	42	51	1.21	-	-	-
	2022–2023	1	1	1	23	36	1.57	-	-	-
	**% change**	**-**	**-**	**-**	**−45%**	**−29%**	**30%**	**-**	**-**	**-**
Panna	2019–2020	10	13	1.3	3,727	8,997	2.41	24	12	0.5
	2022–2023	2	3	1.5	4,113	7,346	1.79	250	131	0.52
	**% change**	**−80%**	**−77%**	**15%**	**10%**	**−18%**	**−26%**	**942%**	**992%**	**4%**
Sagar	2019–2020	15	19	1.27	162	304	1.88	-	-	-
	2022–2023	67	85	1.27	531	1,147	2.16	110	55	0.5
	**% change**	**347%**	**347%**	**0%**	**228%**	**277%**	**15%**	**-**	**-**	**-**
Tikamgarh	2019–2020	9	8	0.89	1694	1826	1.08	-	-	-
	2022–2023	2	2	1	8	13	1.63	-	-	-
	**% change**	**−78%**	**−75%**	**12%**	**−100%**	**−99%**	**51%**	**-**	**-**	**-**

Source: Ministry of Agriculture and Farmer's Welfare (121). % change values are indicated in bold.

In Lalitpur district of Uttar Pradesh and Panna district of Madhya Pradesh, a substantial increase in area and production of small millets is observed. These districts have tribal communities with minor millet-based food systems. Moreover, millets' promotional activities also help to increase the production of small millets in these districts. Region-specific promotional policies for millets revival after IYM, 2023, have helped create awareness of these miracle grains and thereby increased the area of these crops in some districts.

## 5 Growing potential and opportunities of millet cultivation in central India

Millet cultivation is steadily increasing in Bundelkhand due to the crop's ability to adapt to the region's climatic and edaphic conditions. Moreover, large areas of fallow land provide an opportunity to expand cultivation without competing for limited farmland. Furthermore, as tourism in Bundelkhand grows, the local millet industry can benefit by promoting millet-based products, which can generate extra income and help raise awareness of the crop beyond the region. The high nutritional value of millet makes it an ideal crop to fight malnutrition in Bundelkhand, offering a sustainable way to boost both economic stability and food security.

### 5.1 Adaptive traits of millets for Bundelkhand's agroecology

Millets possess multiple complementary adaptations that confer resilience to heat, drought, and degraded soils. From a physiological viewpoint, millets typically have short life cycles (≈12–14 weeks), compact short-statured canopies supported by dense and highly efficient fibrous roots (37). As C4 grasses, millets concentrate CO_2_ around RuBisCO, suppress photorespiration and enhance photosynthetic efficiency at high temperatures (38). This specialized C4 photosynthetic mechanism yields water-use efficiencies up to 1.5 to 4 times greater than those observed in C3 crops, such as rice and wheat (39). In practice, pearl and foxtail millets exhibit “remarkable drought and heat tolerance” along with high nutrient-use efficiency under low-input conditions (40). Seedlings rapidly extend primary roots, far faster than maize/wheat and develop prolific lateral and crown roots (41). These deep roots can penetrate compacted subsoils to extract water. Together, these traits allow millets to thrive on marginal, low-fertility soils with minimal irrigation or fertilizer and can even prosper in acidic, saline, or aluminum-toxic soils (42).

### 5.2 Fallow land as an opportunity for expansion

According to available data, out of the total geographical area of Bundelkhand (7.09 mha), about 61% is arable land, while the rest is designated for various other uses, including 20.45% forest cover (43). Table 9 shows that the region has 4.04 million hectares of net sown area and 7.05 million hectares of gross cropped area. The cropping intensity is 175%, which is considerably lower than the national average (43). The region has 2,074.43 thousand ha under net irrigation, representing only 48% of the total cultivable area and 54% of the net sown area. In contrast, about 2,305 thousand ha area remains rainfed (43), relying solely on rainfall for moisture. The average landholding size is 1.54 hectares, and 77% of landowners own just 39% of the land (44). Persistent water scarcity, a longstanding problem in the region, plays a significant role in its low agricultural productivity. Main crops grown in the cultivated areas include cereals, pulses, and oilseeds. Farmers primarily cultivate food grains, while fruits and vegetables are mostly grown for family consumption, with limited commercialization (45). Since most farmers operate on small plots and grow crops mainly for household needs, thus, farmers often rear small numbers of livestock such as buffaloes, cows, goats, and sheep, which provide vital supplementary income and nutrition for their families.

**Table 9:** Land use pattern in Bundelkhand region of India (2015–16 and 2022–23).

Particulars	Area million (ha) (2015–16)	Area million (ha) (2022–23)
Geographical area	7.09	7.09
Forest	1.42 (20.1)	1.45 (20.45)
Not available for cultivation	0.78 (11.1)	0.75 (10.58)
Other uncultivated land, excluding fallow land	0.50 (7.0)	0.45 (6.35)
Fallow land	0.55 (7.8)	0.22 (3.10)
Net area sown	3.84 (54.1)	4.04 (56.98)
Gross cropped area	5.58	7.05
Areas sown more than once	1.74	3.01
Cropping intensity (%)	145	175

Source: Calculations from data from Directorate of Economics and Statistics, GOI (123).

(Figures in parentheses are the percentage of the total geographical area).

### 5.3 Improved mechanization for higher millet production

Although these traditional grains offer substantial, empirically proven benefits for long-term climate change adaptation and nutritional security, their cultivation and consumption remain limited (46). This is mainly due to challenges such as low yields, high labor demands, costly production, and minimal profits (46, 47). Agricultural mechanization, which involves the systematic use of tools, equipment, and machinery for farming activities, helps replace manual labor with animal power, fossil fuels, or renewable energy throughout the agricultural value chain (48) and (49). Mechanization levels for crops like rice, wheat, maize, sorghum, millets, pulses, oilseeds, cotton, and sugarcane vary widely, with an overall rate of 40% in the country, lower than in comparable developing nations like China (59.5%) and Brazil (75%) (50). Efficient harvesting and threshing processes are vital for millet cultivation, as millets have small seeds that require specialized methods to separate them from mature plants. Developed machinery includes the Multi millet thresher (134); finger millet thresher-cum-pearler (51); multi-millet crop thresher (52); millet thresher (53); axial flow motorized sorghum thresher (54). Furthermore, numerous post-harvest machines have been developed for millet crops, such as the millet mill dehusker (55); foxtail millet dehuller (56); tabletop centrifugal dehuller for small millets (18); finger millet thresher-cum-pearler (57); minor millet dehuller Kodo and Little millet (58); dehulling machines for foxtail and proso millet (59); compact foxtail millet deshelling machine (batch type) (60); barnyard millet dehuller (51); and double-stage dehuller (61).

### 5.4 Millets as an opportunity to combat malnutrition in Bundelkhand

Malnutrition is a significant problem in Bundelkhand, which will be discussed in detail later in this study. Millets, a nutrient-dense crop rich in essential amino acids, vitamins, and a variety of vital micronutrients, can serve as an excellent dietary option to combat this issue in Bundelkhand. By promoting millet consumption and encouraging its cultivation, the region can address food security challenges while providing a sustainable, affordable source of nutrition for its population. The role of millet in improving local diets can greatly impact public health, offering long-term benefits for community wellbeing. Nutri-rich bars or locally produced millets made into “ready-to-use therapeutic foods” (RUTF) can be promoted within local communities to fight malnutrition among the poor. The Central Government has encouraged State Governments and Union Territory Administrations to consider introducing millets into the PM POSHAN Scheme, especially in districts where millet consumption is already a cultural norm (62).

### 5.5 Tourism as a revenue stream for millet products

Bundelkhand, known for its rich history, culture, and natural beauty, has long attracted both Indian and international tourists. Its numerous historical sites, including impressive forts like Orchha, Jhansi, and Kalinjar, as well as the UNESCO World Heritage site of Khajuraho, along with wildlife sanctuaries such as Panna Tiger Reserve, Madhav National Park, and Ken Gharial Sanctuary, have consistently drawn visitors. The region also features many pilgrimage sites such as Chitrakoot, Orchha, and various Jain temples in Khajuraho and Lalitpur, which increase its appeal. As tourism expands in Bundelkhand, there's a great opportunity to promote millet-based products to visitors, generating additional income for local farmers (133). Bundelkhand can highlight millet products as regional specialties, attracting tourists interested in local cuisine. These initiatives can elevate the profile of millet farming in the area and create new income sources, further supporting the region's economy (36, 63).

## 6 Major constraints of millets cultivation in Bundelkhand

**Figure 6:**
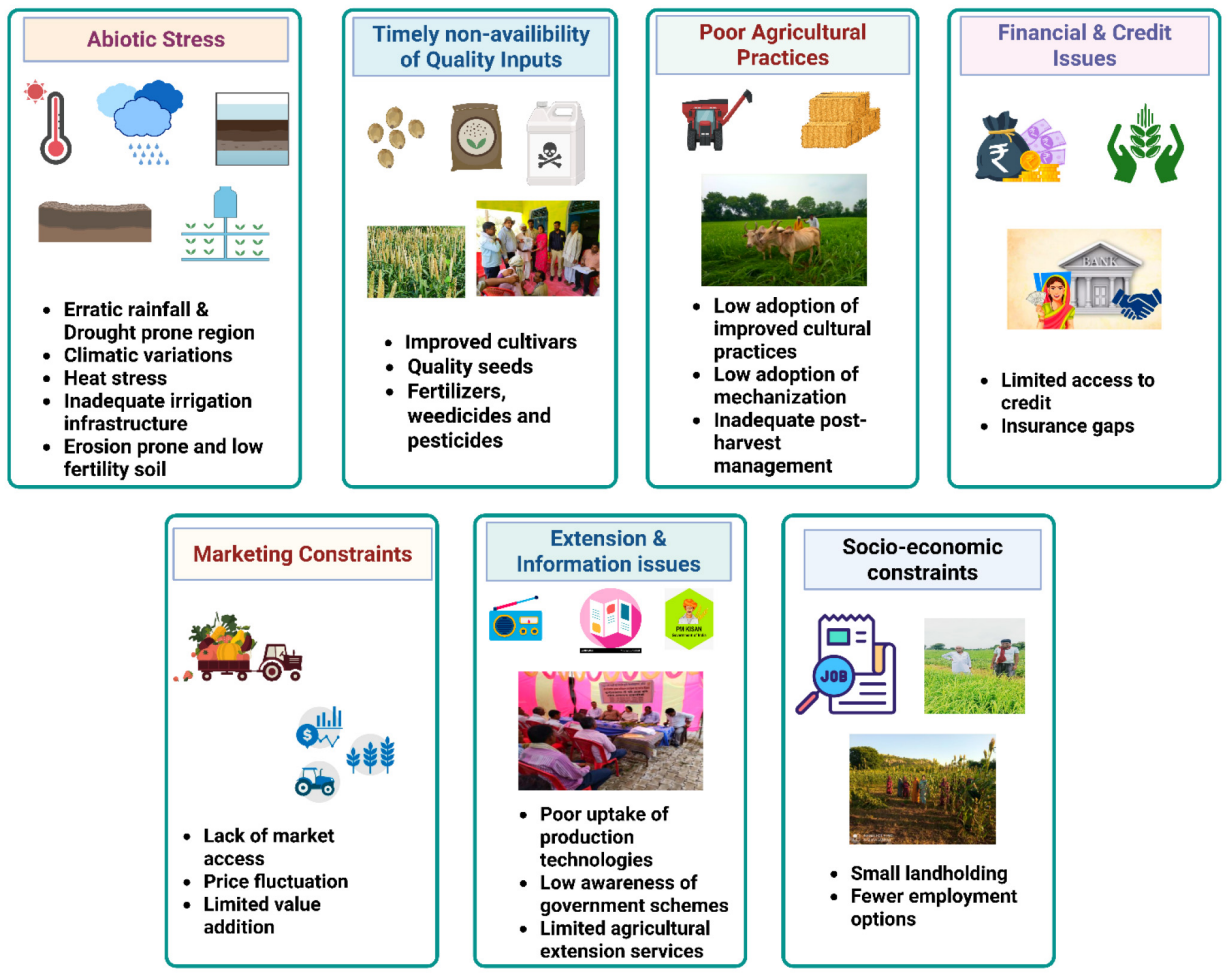
Major constraints of millet cultivation in Bundelkhand; constrains are largely abiotic stresses, delayed or inadequate availability of quality inputs, poor agronomic and post-harvest practices, limited financial and credit access, weak market linkages, inadequate extension support, and smallholder socio-economic limitations.

Millet cultivation in Bundelkhand faces natural, economic, and infrastructural challenges. Tackling these issues will require a collective effort to improve soil and water management, increase awareness of new varieties and cultural practices, and enhance post-harvest management and value addition. It also involves providing improved access to quality inputs, financial support, and market linkages (Figure 6).

### 6.1 Abiotic stress

Millets known for their resilience to climate challenges, but they face various stressor factors during growth that affect their yield, productivity, and usefulness. These stressors are typically categorized as abiotic or biotic types. The primary stresses affecting millet production include drought, waterlogging, crop lodging, and soil salinity.

#### 6.1.1 Erratic rainfall and drought-prone region

The Bundelkhand region is geographically vulnerable to recurrent droughts, unpredictable rainfall, and water scarcity. Meteorological droughts commonly coincide with the onset of the kharif season and may extend through August or September, exposing rainfed crops to prolonged moisture stress during critical stages of growth (7). Such long dry spells cause significant economic losses for local communities, mainly through decreased crop production and yields. Adaptation methods such as crop scheduling, providing supplementary irrigation during these droughts (136), and drought-resistant millet varieties are crucial for reducing the negative effects of climate variability.

#### 6.1.2 Climate variations

Climatic modeling experiments show that temperatures in the Bundelkhand region are expected to rise by approximately 2 to 3.5 °C by the end of this century (64). Furthermore, projections indicate a 5%−10% increase in rainfall by the 2050s, with fewer but more intense rainy days (65). These changes are significant because higher temperatures and uneven rainfall impact millet's productivity. Econometric analysis suggests that a 1% increase in the annual average temperature results in a 3.06% decrease in millet yield (6).

#### 6.1.3 Heat stress

The Bundelkhand region has a hot, semi-arid climate with large temperature fluctuations. Summer temperatures frequently exceed 40 °C, especially in May and June, while winter temperatures can drop to as low as 1 °C in December and January. The average annual temperature is around 25 °C, with the hottest months in late spring and the coldest in winter. Such extreme temperatures during flowering or grain filling can disrupt pollination and grain development, resulting in poor yield.

#### 6.1.4 Inadequate irrigation infrastructure and depleting groundwater

Water scarcity is a defining characteristic of the dryland ecosystem (135) in Bundelkhand, where only a small fraction of the agricultural land is irrigated. The majority of cultivation in this region remains rainfed and highly dependent on the erratic and uneven distribution of monsoon rainfall (51). Long-term rainfall data from 23 stations in Uttar Pradesh, part of the Bundelkhand region (seven districts), show a 200 mm decline in annual rainfall over six decades, increasing the region's vulnerability (66–68). The persistent, threats of recurrent droughts, prolonged dry spells, and uncertainty in seasonal rainfall patterns make local farmers highly hesitant to risk capital by planting *kharif* crops. Consequently, they typically cultivate only a single crop during the rabi (post-monsoon) season, relying on residual moisture or supplemental irrigation (68, 69). The traditional practice of decentralized rainwater harvesting, known as the haveli system, has recently become ineffective due to social apathy and neglect (68, 70). About 70%−80% of the region depends on shallow groundwater systems for farming and household use, which are under stress because these wells function for only a few months (68, 71). Farmers typically follow calendar-based irrigation schedules and still use traditional flood irrigation methods, which have low distribution efficiency and require more energy (66).

#### 6.1.5 Erosion-prone and low-fertility soil

In general, Bundelkhand soils can be classified into four types based on their properties: rakar (17.6%), parua (38.5%), kabar (31.4%), and mar (12.4%). Rakar soils are coarse-textured, shallow, and highly permeable, whereas parua soils are alluvial, consisting of a mixture of red and brown soils with moderate infiltration rates. Mar and kabar are black soils that extend to a depth of 1 m, and are characterized by fine texture, having swelling and shrinking properties, and crack development during summer (5). Predominating red soils are newly formed, contain gravels, and are quite shallow (4). These soils typically have poor moisture retention and cannot support rabi crops solely on residual moisture.

### 6.2 Availability of quality inputs on time

Seed, fertilizer, and crop protection products, along with seedlings, feeds, and machinery that support crop and allied production, are examples of agricultural inputs (72). Agricultural inputs are external resources improve crop performance and productivity, ranging from high-quality seeds to advanced machinery like tractors. Indian farmers are characterized by small and fragmented land holdings, dependence on rain for irrigation, use of traditional farming practices, involvement in subsistence farming, and lack of resources, education, and media access (73–75). Agricultural markets are still underdeveloped and lack proper infrastructure and processes (76). The major constraints are:

#### 6.2.1 Improved cultivars

Globally, millets have received very limited research on developing plant varieties to improve yield traits and resistance to biotic and abiotic stress (9). The level of research has historically been low for millets compared to wheat, rice, and maize (77). There is a lack of improved cultivars for this region and no dedicated center to develop cultivars that meet the needs of Bundelkhand farmers. The extensive germplasm collections of millets, which include over 138,000 accessions preserved at the International Crops Research Institute for the Semi-Arid Tropics (ICRISAT) and the National Bureau of Plant Genetic Resources gene banks, serve as a valuable resource for genetic improvement of millet crops. Adequate genetic variation has been reported for various agronomic, adaptation, and grain quality traits (78, 79).

#### 6.2.2 Quality seeds

Many farmers rely on low-quality or saved seeds, leading to reduced productivity and higher vulnerability to diseases due to limited access to improved, high-yield millet seed varieties. The lack of investment in research and development remains a key barrier to the global expansion of millet cultivation. Despite their nutritional benefits and resilience in harsh environments, millets face challenges such as slow technological progress, low-yield varieties, and weak market infrastructure, which hinder their widespread adoption and commercialization. The Agriculture and Allied Activities Sector accounts for about 16% of the country's GDP for FY24 (PE) (80). This sector remains essential to national income, employment (supporting approximately 46.1% of the population), food security, and economic growth, and it affects other sectors of the economy. Funding for agricultural research and development through DARE has increased only slightly by 0.65%, to Rs. 9,941 crore for 2024–25 from Rs. 9,876 crore the previous year. This amounts to roughly 0.2% of the agricultural gross domestic product (GDP), which is about Rs. 47 lakh crore at current prices in 2023–24. This is markedly lower than in other major emerging economies like China and Brazil, which allocate 0.6% and 1.6%, respectively (81). These limited funds restrict efforts to develop improved millet varieties and limit the availability of high-quality seeds (8).

#### 6.2.3 Fertilizers, weedicides, and pesticides

Timely and affordable access to fertilizers, pesticides, and herbicides is crucial for establishing healthy crops. Having these inputs available at key stages of crop growth can help prevent losses in millet yields. Marginal farmers more often face these challenges due to limited credit, which hinders their ability to invest in their farms and increase productivity (82).

### 6.3 Poor agricultural practices

#### 6.3.1 Low adoption of improved cultural practices

Since independence, Indian agricultural policies have consistently aimed to improve farming infrastructure, promote better agricultural practices, and support the socio-economic development of farming communities. Despite a significant rise in agricultural output over the years, many farming communities in India still face poverty (83). The economic unsustainability of the crop production sector, especially for small and marginal farmers, is causing an agrarian crisis that could negatively impact the future of agriculture in the country (84). Educating farmers on the efficient use of agricultural inputs and encouraging the adoption of improved farming techniques can greatly cut crop production costs. In areas like Bundelkhand, initiatives such as accelerated seed replacement—substituting varieties older than 10 years with new ones—and incentivizing the public sector while enabling the private sector to boost quality seed production will be crucial to increasing crop output per unit of time and space (85). Farmers should also be encouraged to follow recommended agronomic practices and ensure proper calibration and balanced use of various agricultural inputs.

#### 6.3.2 Low adoption of mechanization

Challenges in this area include a lack of awareness about new machines, limited availability of equipment designed for millet cultivation, inadequate financial support for adopting new technologies, and insufficient training of extension workers in machine functionality and compliance with standards (86). The use of agricultural machinery such as tractors, planters, and harvesters is low in Bundelkhand due to high costs and limited access to credit, leading to labor-intensive and less efficient farming.

#### 6.3.3 Inadequate post-harvest management

India faces significant grain storage losses exceeding 14 million tons annually (87). Millets are no exception; they are vulnerable to spoilage if not stored at proper moisture levels in adequate storage facilities. Inadequate post-harvest technologies for value addition, along with a lack of awareness, knowledge, and skills among farming communities regarding processing technologies to add value, further diminish the potential to maximize profits from the harvest (88, 89).

### 6.4 Financial and credit issues

#### 6.4.1 Limited access to credit

Credit is undoubtedly a vital input, acting as a catalyst for development. However, it can only supplement, not replace, other factors. India's role in the rural credit mechanism mainly involves working through commercial banks, rural regional banks, and cooperative banks. This limits their capacity to invest in better inputs and technologies (10). Access to institutional credit is higher in the western district than in the Bundelkhand region of Uttar Pradesh (45). The total institutional credit to the agricultural sector increased from Rs. 39,305 crores in 2015–16 to Rs. 169,585 crores in 2022–23 in Uttar Pradesh, and from Rs. 3,952.24 crores to Rs. 9,451.25 crores in Bundelkhand during the same period. The credit disbursed per hectare of Gross Cropped Area (GCA) was highest in Jhansi at Rs. 64,870 and lowest in Chitrakoot at Rs. 11,026 (10). In 2022–23, the highest investment credits in the Bundelkhand region went to Hamirpur (58.44%), Jalaun (23.75%), and Lalitpur (5.72%). Conversely, Banda (0.45%), Chitrakoot (2.59%), and Mahoba (4.54%) received the lowest shares of investment credit. These district disparities in outreach may stem from differences in resource endowments and technological adoption. Based on disbursement data, commercial banks provided the largest share of credit (51.88%), followed by RRBs (33.66 %) and cooperative banks (13.92%) in 2021–22 in Bundelkhand. Agricultural credit disbursed per hectare of GCA was Rs. 31,833 in 2022–23. Notably, the highest per-hectare credit was in Jhansi at Rs. 64,870, followed by Jalaun at Rs. 46,146, Hamirpur at Rs. 37,550, and Lalitpur at Rs. 27,837. Conversely, the lowest credits were in Chitrakoot at Rs. 11,026, Mahoba at Rs. 12,840, and Banda at Rs. 13,722.

#### 6.4.2 Insurance gaps

Agricultural insurance is widely recognized as a crucial tool for helping farmers and herders mitigate some of the negative financial consequences of adverse natural events (11). Clearly, many countries have used insurance to manage agricultural risks (90–92). However, the adoption of crop insurance in Bundelkhand remains limited. Farmers often shoulder the losses from crop failures caused by drought, pests, or disease without sufficient compensation from the government.

### 6.5 Marketing constraints

Limited access to markets is a major obstacle in establishing millets as an alternative to major cereals. Farmers in Bundelkhand often sell their produce to local traders at lower prices due to small quantities and transportation issues. This monopoly causes high price fluctuations. The solution to limited market access is seen in adding value to primary produce, but the lack of processing infrastructure makes it just wishful thinking.

### 6.6 Extension and information issues

The main reasons for the low adoption of modern production techniques in millet farming in Bundelkhand are poor extension services, lack of farmer education, training programs, and incentive schemes. The absence of these factors prevents farmers from learning about government programs that could help them, such as subsidies for irrigation equipment, improved seeds, or financial aid.

### 6.7 Socio-economic constraints

Most farmers in Bundelkhand are small or marginal, owning less than two hectares of land. This limits their ability to invest in expensive inputs and technology, making them more vulnerable to crop failures. Due to limited employment opportunities, many families in Bundelkhand migrate to cities in search of work, leaving fewer members to manage the farm, which hurts agricultural productivity.

## 7 R & D scenario of millets in India

Millet research in India started early in the 20th century. Pre-independence systematic breeding of finger millet began at Zonal Agricultural Research Station, V.C. Farm, Mandya, in 1913 under Leslie C. Coleman, enabling the development of pure line cultivar H 22 in 1918. Following, independence, breeding work continued under coordinated research programs, in 1956 the ICAR started the Project for Intensification of Regional Research on Cotton, Oilseeds, and Millets (PIRRCOM) at 17 centers, several focused on sorghum and pearl millet neglecting others, possibly the Indian millet research strategy was influenced by the success of CMS based sorghum and pearl millet hybrids in the United States (93). The All-India Coordinated Sorghum Improvement Project was established in December 1969 with the primary goal of enhancing grain and forage sorghum through multiple collaborating centers nationwide. The regional station of the Indian Agricultural Research Institute (IARI) in Hyderabad was transformed into the National Research Center for Sorghum (NRCS) in 1987, which later merged with the All-India Coordinated Sorghum Improvement Project (AICSIP). In 2009, NRCS was upgraded to the Directorate of Sorghum Research (DSR), which expanded in 2015 into the Indian Institute of Millets Research (IIMR) to focus on all millet crops. It was proposed in 2023 to develop it as a center of excellence (94). The All India Coordinated Millet Improvement Project (AICMIP) was established in 1965. In 1985, pearl millet was separated from other millet crops, leading to the formation of the All India Coordinated Pearl Millet Improvement Project (AICPMIP). Since then, AICPMIP has led the development of diverse improved breeding lines, hybrids, and parental lines, playing a pioneering role in millet crop enhancement (67).

The coordinated breeding efforts have yielded hundreds of improved varieties and hybrids. To date (2014–2023), the Central Variety Release Committee has approved 229 millet varieties for diverse ecologies: 67 sorghum, 68 pearl millet, 19 little millet, 11 kodo, 10 foxtail, 7 proso, 6 barnyard, 40 finger millet, and 1 brown top millet (95). Substantial improvements have been made in sorghum and pearl millet, with over 100 cultivars developed under the AICRP system. Numerous hybrids producing 30%−50% higher yields were introduced for both crops. Successful hybrid development in these mostly cross-pollinated crops was facilitated by the availability of a male sterility system for hybrid seed production. These hybrids have increased yield potential, reaching 5–7 tons per hectare under irrigation in summer. Similarly, in Finger millet, early improvement by recombination breeding among Indian and African ecotypes resulted in 16 Indo-African varieties designated as “INDAF” series, resulting in an increase in productivity (2.5 quintalhec-1) with 50% increase in Karnataka (96). Male sterility sources have been identified in finger millet, such as the GMS line INFM 95001; their utilization in hybrid breeding has remained limited (97). Unlike sorghum and pearl millet, where cytoplasmic male sterility (CMS) systems facilitate large-scale hybrid seed production. Conversely, the small millets (little, foxtail, proso, barnyard, kodo) have lagged in varietal development. Breeding has been constrained by a narrow genetic base, the absence of sterility systems (98), and insufficient funding for improvement programs. While some varieties exist, most are based on pure line selection and hybridization among diverse ecotypes.

## 8 Status of malnutrition and biofortified varieties

Malnutrition results from factors at the individual, household, and community levels (99). Child malnutrition is often linked to poor food quality, inadequate food intake, and severe, recurrent infectious diseases, or combinations of these factors (100, 101). According to the United Nations Children's Fund (UNICEF), stunting, or low height for age, causes irreversible physical and mental harm to children (102). Child health and nutritional development require a comprehensive approach involving multiple sectors to fight malnutrition among children (103–106). However, under-resourced areas or underdeveloped regions with complex socio-demographic factors may also contribute to child malnutrition (99, 107, 108). Studies show significant economic disparities in children's health status and the utilization of Integrated Child Development Scheme (ICDS) services in India (107, 109–111). Although nutrition is crucial for achieving Sustainable Development Goals (SDGs) in any country (112), it remains an underprioritized and neglected sector in India. This sector, encompassing nutrition and related areas such as food security, agriculture, water and sanitation, health practices, gender, and health communities, is advocating strongly for nutrition within the SDGs framework (92, 107, 113). India has yet to effectively improve the nutritional health of children, particularly issues related to underweight, wasting, and stunting.

**Figure 7:**
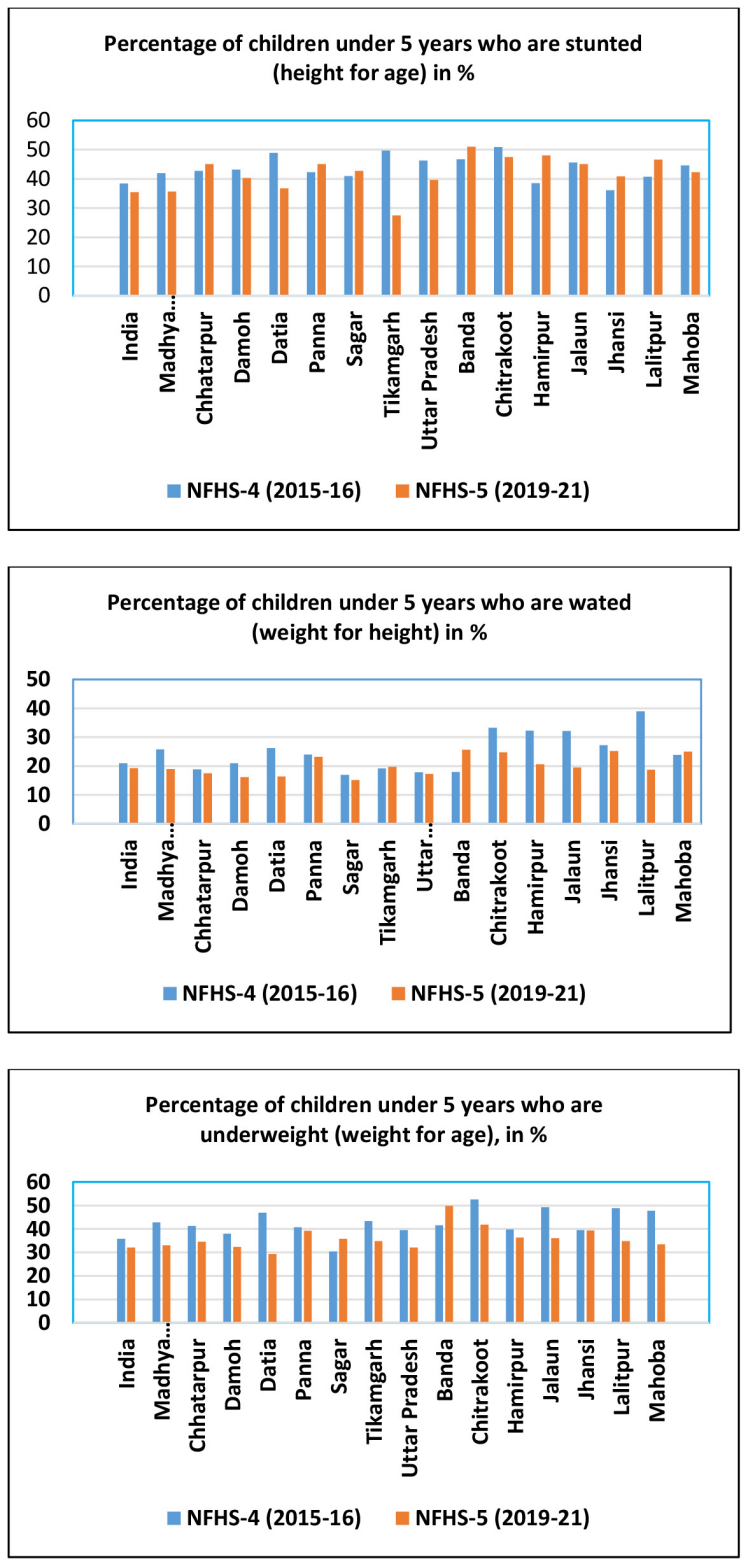
Comparative status of child malnutrition indicators in India and Bundelkhand districts based on NFHS-4 (2015–16) and NFHS-5 (2019–21). The figure presents district-wise changes in the percentage of children under five years who are stunted, wasted, and underweight across selected districts of Uttar Pradesh and Madhya Pradesh.

Malnutrition is a serious issue in Bundelkhand. Across 13 districts in Bundelkhand, the proportion of children under 5 who are stunted has increased between 2015–16 and 2020–21. The recently released data from the National Family Health Survey (NFHS), 2020–21, show that nationwide, the percentage of children under 5 who are stunted decreased from 38.4% to 35.5% between 2015–16 and 2019–21, a decline of 2.9% points (Figure 7). In Madhya Pradesh, the percentage of children under 5 who are stunted decreased from 42.0% to 35.7% between 2015–16 and 2020–21, a reduction of 6.3% points. In Uttar Pradesh, the same metric dropped from 46.3% to 39.7% during the same period, a decrease of 6.6% points (Figure 7) (114).

Indian NARES (National Agricultural Research and Education System), along with ICRISAT, is leading the development of biofortified millet varieties. Its biofortification breeding program for pearl millet follows a three-phase strategy: Phases I, II, and III. The first phase is a short-term approach focused on trait genetics, germplasm screening, and the generation of genetic diversity. The second phase is a medium-term approach that involves validating high-iron and zinc breeding lines and hybrid parents identified through conventional breeding to rapidly develop biofortified varieties or hybrids. The third phase is a long-term approach that develops breeding lines rich in iron and zinc, along with hybrid parent varieties, while maintaining genetic diversity through the ongoing integration of micronutrient traits (115). Collaborative efforts between national and international programs have led to the development of 12 millet varieties: 8 pearl millet, 3 finger millet, and 1 small millet. The finger millet varieties CFMV 1 and 2 are high in calcium, iron, and zinc, while the small millet variety CCLMV1 has elevated levels of iron and zinc (Table 10). Additionally, the Extension Division of ICAR has launched two special programs—Nutri-sensitive Agricultural Resources and Innovations (NARI) and Value Addition and Technology Incubation Centers in Agriculture (VATICA)—to promote the adoption of biofortified varieties through its Krishi Vigyan Kendras (KVKs) (94).

**Table 10:** Recently released high-yielding biofortified millet varieties and hybrids with enhanced nutritional traits.

Crop	Hybrid/variety	Grain yield (kg/ha)	Year	Bio-fortified elements	Recommended states	References
Pearl millet	Dhanashakti	3,000–3,200	2014	Iron, Zinc	Rajasthan, Maharashtra, Gujarat	(115, 124–126)
	Shakti 1201	2,500–2,800	2018	Iron, Zinc	Haryana, Gujarat, Madhya Pradesh	(115, 126)
	Siri 1253	3,000–3,500	2021	Iron, Zinc	Rajasthan, Haryana, Gujarat	(115, 126)
Sorghum	Parbhani Shakti ICSR 14001	4,000	2018	Iron Zinc	Madhya Pradesh, Karnataka, Telangana, Maharashtra	(127)
Foxtail millet	CO 15	1,600–1,800	2019	Iron, Zinc	Karnataka, Tamil Nadu, Maharashtra	(128)
	DHFT 109-3	2,800–3,000	2020	Iron, Zinc	Gujarat, Rajasthan, Uttar Pradesh	(88)
Kodo millet	TNAU 86	1,800–2,000	2017	Calcium, Iron, Zinc	National	(117)
Finger millet	PRM-1	2,000–2,200	2016	Iron, Zinc	Odisha, Chhattisgarh, Jharkhand	(129)
	CFMV 1 (Indravathi)	3,110	2020	Calcium, Fe, Zn	Andhra Pradesh, Karnataka, Tamil Nadu, Puducherry, Odisha	(130)
	CFMV 2 (Gira)	2,950	2022	Calcium, Fe, Zn	Andhra Pradesh, Chhattisgarh, Gujarat, Maharashtra, Odisha	(130)
Little millet	CLMV 1 (Jaicar Sama-1)	1,580	2021	Fe, Zn	Maharashtra, Andhra Pradesh, Telangana, Tamil Nadu, Puducherry	(130)

## 9 Strategies for revival of millets in Bundelkhand

Bundelkhand, once a prosperous region for growing various millet types, faced a significant decline in millet farming due to the adoption of new agricultural methods and crops. The effort to revive millet farming in Bundelkhand is gaining momentum, supported by initiatives from the central and state governments. The plan for millet revival in Bundelkhand focuses on improving farming techniques, expanding market links, and leveraging the region's climate for millet cultivation.

**Figure 8:**
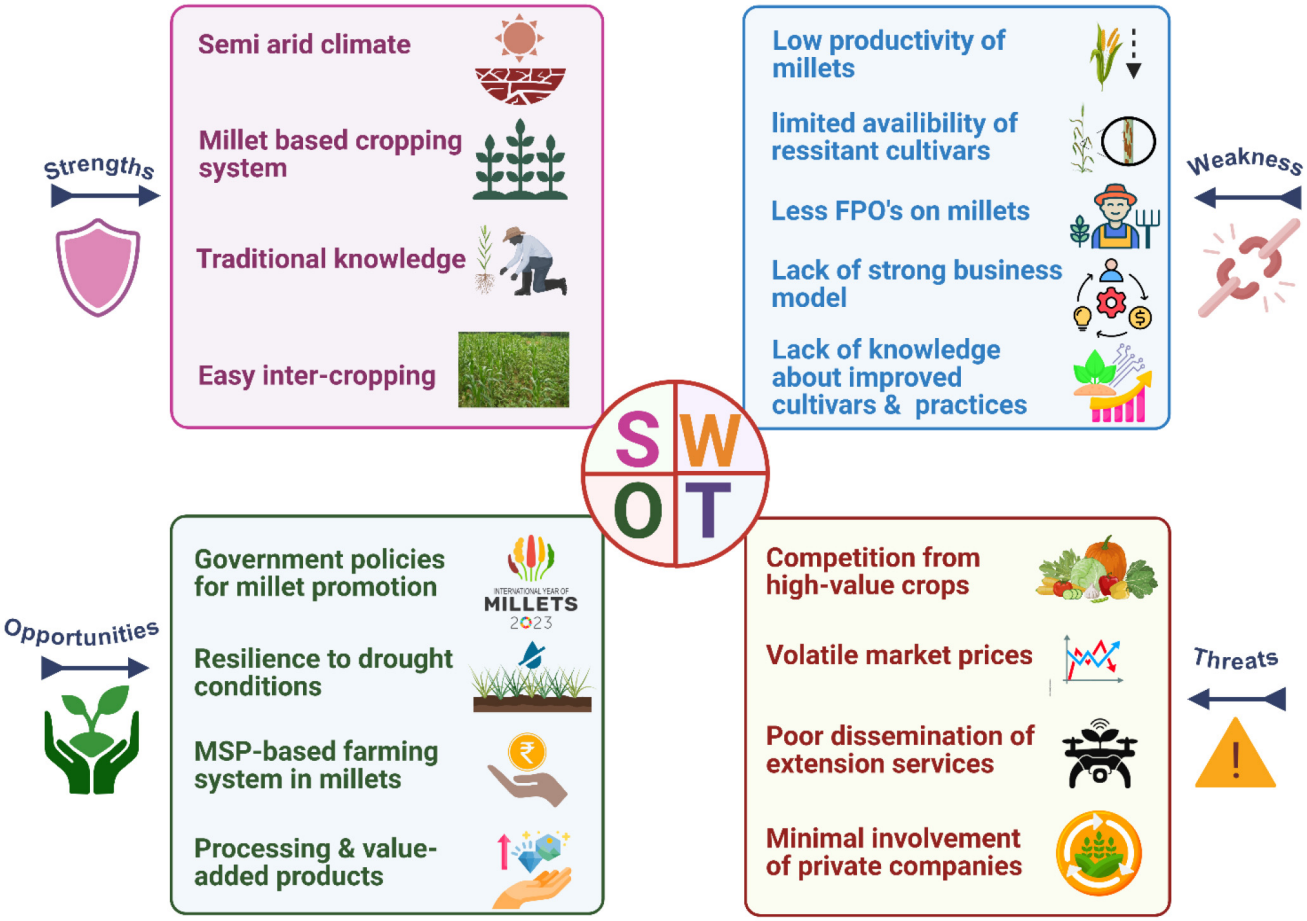
SWOT framework for millet production and value-chain development in Bundelkhand, India.

The strengths, weaknesses, opportunities, and threats (SWOT) of millet cultivation in Central India are presented in Figure 8.

The revival process involves strategic interventions across various fronts.

### 9.1 Policy support

The Government of India celebrated the year 2023 as the “International Year of Millets” to turn it into a people's movement, promoting Indian millets, recipes, and value-added products worldwide. Millets gained prominence during India's G20 presidency through events like the Millet Culinary Carnival, international trade shows, a chef's conference, exhibitions by Farmers Producer Organizations (FPOs), roadshows, kisan melas, chef training for paramilitary forces, and the ASEAN India Millet Festival in Indonesia. To establish India as a global hub for “Shree Anna,” the Indian Institute of Millets Research (IIMR) in Hyderabad has been designated as the Global Center of Excellence for sharing best practices, research, and technology at both national and international levels (95). Additionally, the new Regional Research Center for Bajra in Gudamalani, near Barmer, Rajasthan, was inaugurated on September 27th, 2023. To enhance global research collaboration and public awareness of millets, a new initiative named “Millets and OtHer Ancient GRains International ReSearcH Initiative (MAHARISHI)” has been launched during the G20 Presidency. Such promotional policies need to be continued to support millet cultivation.

Similarly, the Union Cabinet approved the increase in the Minimum Support Prices (MSP) for all millets for the marketing Season 2024–25. Pearl millet observed an increase of Rs. 125 over the previous year, with an MSP of Rs. 2,625 q. Sorghum hybrid (3,371 Rs/q) and sorghum maldandi (3,421 Rs/q) had increased by Rs, 191 and 196, respectively, from last year. Finger millet observed the highest increase in MSP of RS 444 with MSP of Rs. 4,290 Rs/q (30).

### 9.2 Tailoring new generation varieties and hybrids

For Bundelkhand, millet improvement breeding programs, should be focused on region-specific plant ideotypes; combining drought tolerance, early maturity, stable yield, efficient canopy architecture, biotic-stress resistance, and improved grain nutritional quality. Such ideotypes can be developed more efficiently through the integration of conventional breeding with high-throughput phenotyping, omics-assisted trait discovery, genomic selection, and genome editing (116).

### 9.3 Crop production

This step involves adopting agronomic practices that are resilient to climate change, specifically tailored to the semi-arid conditions of the region. Emphasizing standardization of sowing dates, intercropping, cropping systems, and low-input sustainable farming techniques will be crucial for enhancing millet productivity while preserving soil health. Weeds are a major problem for millets, as no recommended herbicides are present, particularly for minor crops. Research efforts should be focused in these directions.

Nutritional profiling of soils in Bundelkhand will help develop biofortified varieties rich in iron, zinc, and calcium, ensuring they not only survive but also thrive in local soil conditions and provide higher nutritional value to consumers.

### 9.4 Crop protection technologies

This step involves incorporating crop protection methods that reduce biotic stresses. Developing affordable, eco-friendly pest and disease management solutions specifically for millet crops will help maintain these crops without imposing additional costs on farmers.

### 9.5 Millets distribution

Millets can also be distributed through the public distribution system (PDS), although only a few states currently supply them through ration shops. The PDS can be strengthened by emphasizing the high nutritional quality of these millets across the country (117).

### 9.6 Processing

Secondary and tertiary processing, including milling, puffing, and incorporating millets into ready-to-eat products, will further add value and expand market opportunities, leading to higher profits for farmers and entrepreneurs in the region. There is a need to develop and provide suitable machinery and training for farmers.

### 9.7 Development of millet clusters and support systems

Creating seed hubs for these millets will ensure access to high-quality seeds, increase seed output, and develop a sustainable supply chain. Establishing buyback agreements between farmers and local processing units can ensure market stability and provide a consistent income.

### 9.8 Entrepreneurship and marketing

Millet-based products face significant challenges in Bundelkhand due to limited market access, weak supply chains, and low consumer demand. To address these issues, entrepreneurship hubs should focus on developing value-added products such as millet flour, snacks, and beverages, while encouraging youth and women to enter agribusiness. Establishing processing units will improve product shelf life and marketability, and targeted marketing campaigns can raise awareness of millet's nutritional benefits and connect Bundelkhand's farmers to a broader market. The journey of millet revival is not just about reintroducing a forgotten crop; it is about restoring ecological balance, supporting farmers' livelihoods, and nourishing future generations. In Bundelkhand, resilient millet will rise again stronger and more relevant than ever.

## 10 Conclusion

Millets can provide both climate resilience and nutritional security to climate-vulnerable regions like Bundelkhand. However, over the years, the region has experienced a significant decline in millet cultivation, often driven by shifts toward input-intensive monocropping or biases in government procurement policies. The analysis presented here emphasizes that reviving millets in Bundelkhand cannot rely solely on crop attributes; instead, strategic interventions are necessary to overcome barriers in seed system extension services, market linkages, credit access, and crop insurance. In addition to investments in processing and mechanization, adding value will be essential to increase profitability. We also recommend incorporating millets into the public distribution system and midday meals to connect production with demand. The SWOT analysis results reveal sufficient opportunities for Bundelkhand to establish itself as a hub for climate-smart, nutrient-rich grains, provided weaknesses and threats are systematically addressed. Reviving millet farming will not only diversify cropping systems but also improve food security, decrease vulnerability to climate variability, and generate livelihood opportunities through agri-entrepreneurship and tourism-driven value chains.
